# Tribological, Mechanical and Thermal Properties of Fluorinated Ethylene Propylene Filled with Al-Cu-Cr Quasicrystals, Polytetrafluoroethylene, Synthetic Graphite and Carbon Black

**DOI:** 10.3390/polym13050781

**Published:** 2021-03-04

**Authors:** Leonid K. Olifirov, Andrey A. Stepashkin, Galal Sherif, Victor V. Tcherdyntsev

**Affiliations:** Laboratory of Functional Polymer Materials, National University of Science and Technology “MISIS”, Leninskii Prosp, 4, 119049 Moscow, Russia; m80786@yandex.ru (L.K.O.); a.stepashkin@misis.ru (A.A.S.); eng_galal_emad@mu.edu.eg (G.S.)

**Keywords:** composites, fluoroplastic, quasicrystals, polytetrafluoroethylene, ball milling, injection molding, wear resistance, coefficient of friction, thermal conductivity

## Abstract

Antifriction hybrid fluorinated ethylene propylene-based composites filled with quasicrystalline Al_73_Cu_11_Cr_16_ powder, polytetrafluoroethylene, synthetic graphite and carbon black were elaborated and investigated. Composite samples were formed by high-energy ball milling of initial powders mixture with subsequent consolidation by injection molding. Thermal, mechanical, and tribological properties of the obtained composites were studied. It was found that composite containing 5 wt.% of Al_73_Cu_11_Cr_16_ quasicrystals and 2 wt.% of nanosized polytetrafluoroethylene has 50 times better wear resistance and a 1.5 times lower coefficient of dry friction comparing with unfilled fluorinated ethylene propylene. Addition of 15 wt.% of synthetic graphite to the above mentioned composition allows to achieve an increase in thermal conductivity in 2.5 times comparing with unfilled fluorinated ethylene propylene, at that this composite kept excellent tribological properties.

## 1. Introduction

Fluorinated ethylene propylene (FEP) is a high-temperature semicrystalline thermoplastic capable of working at temperatures up to 200 °C, with favorable properties that include high thermal stability, flammability, chemical resistance, dielectric strength, low water absorption and low melt viscosity. The structure of FEP is similar to that of polytetrafluoroethylene, except that a trifluoromethyl group is introduced along the side of the polymer main chain. The molecular weight of FEP is much lower than that of polytetrafluoroethylene, which results in significantly lower melt viscosity and better processability [1]. FEP is difficult to wet with organic liquids and exhibits low friction coefficients. Fluoropolymers are extensively used in polymer/metal tribological systems operating in dry, mixing and wet environments [2]. About 65% of overall worldwide consumption for FEP is in plenum cable insulation. 

The application of unfilled FEP has been limited because of its low mechanical strength, low wear resistance and high coefficient of thermal expansion. FEP-based blends [3] are good candidates for injection or compression molding applications requiring elevated temperature resistance, wear resistance, low friction, excellent chemical resistance, and toughness. The use of various fillers in fluoropolymers improves their rigidity, dimensional stability, reduced mold shrinkage, creep, lowered coefficient of thermal expansion, increased thermal conductivity and wear resistance [4]. However, in contrast to polytetrafluoroethylene, little data on the FEP-based composites reinforced with inorganic fillers is found, all related to various carbon materials. Fillers such as graphite [5,6], graphene [7] and carbon nanotubes [8] were utilized in FEP-based composites. Additionally, combined carbon fillers, namely graphite + carbon nanotubes [9] and graphite + carbon fibers [10] were applied as FEP reinforcement. It was observed that FEP filling with carbon materials improve the processability of FEP [8] and the mechanical properties [7,8], and allows one to obtain composites with high electrical [5,6,9] and thermal [10] conductivities. An increase in the thermal conductivity is of particular importance for tribological applications because it can contribute to a reduction in heat development in the sliding contact area [11].

Due to their good processability, FEP-based composites are suitable candidate materials for bearing and sealing applications in pumping equipment and automotive technology. Such applications require high antifriction behavior, including good wear resistance, low friction coefficients and low wear of the counterbody. We propose to optimize the antifriction properties of FEP using combination of fillers, as the reinforcing of polymer matrix with hard particles allows one to improve the wear resistance. As it also decreases the friction coefficient and provides self-lubrication properties, the addition of polymer with low friction coefficient and/or of solid lubrication materials with graphite-like crystalline structures is required. 

Among a number of inorganic fillers used to improve the antifriction properties of polymers, quasicrystals (QCs) are of particular interest. QCs are a family of intermetallic compounds possessing crystal lattices with symmetry axes forbidden by classical crystallography. QCs have good antifriction properties, because they combine high hardness and very low friction coefficients. This low friction coefficient of QCs follows from their low surface energy (28 mJ/m^2^) [12]. In recent years various polymers were studied as a matrix for QC-reinforced composites. 

In [13,14,15,16] epoxy resin-based composites filled with QC were investigated. It was noted that QC significantly increase the wear resistance of the epoxy resin [13,14], whereas nearly no effect of QC content on the friction coefficient value was observed [14]. An important advance of QC fillers consists in minimizing the abrasion of steel counterfaces [13]. It was noted that QC incorporation results in no significant loss of epoxy resin adhesive properties [15].

Most investigations on QC-reinforced polymers were carried out using thermoplastic polymers as matrix materials. In [17] linear low-density polyethylene-based composites were studied, and it was found that besides increasing the mechanical properties and wear resistance, filling of this polymer with QC results in a significant decrease in the friction coefficient in relation to pure matrix polymer. A positive effect of high density polyethylene reinforcement with QC was observed in [18], where an increase in polymer wear resistance as a result of filling was accompanied with no abrasion of an aluminum counterface. Several investigations on QC-filled ultra-high molecular weight polyethylene were carried out [19,20,21,22,23]. Most of them confirm the positive effect of the QC reinforcement on the polymer frictional behavior; however, in [23] the chipping of QC particles from the polymer matrix was reported. Such chipped particles cause abrasive wear of the studied composite and counterface, and a significant increase in the friction coefficient volume. The same effect of QC filler was observed in the multicomponent polypyromellitimide- based composites [24]. Chipping may occur due to weak chemical bonding between the QC fillers and the polymer matrix. To create chemical bonding between QC and polymers surfactant treatment of QC particles can be used [20,25].

Besides polyethylenes, various thermoplastic polymers, such as polyamide [26,27], polyaniline [28], polyphenylene sulfide [29,30] and polyaryl ether ketone [30] were studied as matrices to form QC-reinforced composites. The obtained composites show behaviors close to those discussed above. Filling of polymers with QC results in the significant increase of the mechanical properties [29] and wear resistance [27,30]. As it was shown in [26], polyamide reinforced with QC exhibits higher wear resistance and lower friction coefficients in comparison not only with pure polyamide, but also with polyamide filled with pure Al, carbon and glass fibers and glass particles.

Fluoropolymers reinforced with QC was also reported in several papers. Polytetrafluoroethylene- [31,32] and ethylene-tetrafluoroethylene [33]-based composites reinforced with QC were investigated. It was noted [32] that in contrast to the ultra-high molecular weight polyethylene-based composites, tested under the same conditions [23], a positive effect of QC on the wear resistance of polytetrafluoroethylene and ethylene-tetrafluoroethylene-based composites was observed, which was associated with better QC adhesion to fluoropolymers than to polyethylenes. This indicates that fluoropolymers should be considered as suitable matrices for QC-filled composites. In [34] FEP-based multicomponent composites were investigated, and it was observed that addition of QC particles significantly increases the wear resistance and decreases the friction coefficient of composites.

It should be noted that in most of composites discussed above icosahedral QC alloys of a Al-Cu-Fe [13,15,17,19,20,21,23,24,29,30,31,32,33,34] system or of Al-Cu-Fe systems alloyed with boron [14,16,26] were used as reinforcing particles. Indeed, Al-Cu-Fe is the most investigated QC-forming system, as the icosahedral QC phase in this system is thermodynamically stable, easy to synthesize, and possesses a number of advanced properties [35]. However, from the viewpoint of tribological applications of polymer composites, including applications in aggressive environments, the corrosion resistance of fillers should be taken into account. It is known that Al-Cu-Fe QC phases does not possess excellent corrosion resistance, and no evidence for improvement in corrosion resistance due to QC structures was observed for Al-Cu-Fe alloys [36]. Corrosion studies of Al-Cu-Fe alloys of various compositions show that a decrease in Fe content results in an increase in the corrosion resistance [37]. As it was shown in [38], replacing of Fe with Cr in QC-containing Al-Cu-Fe-Cr alloys results in significant increases in the corrosion resistance. That is why it is reasonable to use Al-Cu-Cr QC particles as fillers in polymer composites for tribological applications. It should be noted that in contrast to Al-Cu-Fe systems, where an icosahedral QC phase is formed, Al-Cu-Cr systems more often form decagonal QC phases; however, as it was shown in [18], the decagonal QC of Al-Ni-Co systems shows excellent behavior as a filler for polymer matrices, and we can expect the same behavior from Al-Cu-Cr decagonal QC. Recently we investigated the formation of decagonal Al-Cu-Cr QC powder by mechanical alloying with subsequent annealing [25,39]. In the present study the synthesized powders were applied as hard fillers to improve the wear resistance of antifriction FEP-based composites. 

To decrease the friction coefficient, we decided to use a small addition of polytetrafluoroethylene (PTFE) because it possesses the lowest coefficient of friction among fluoropolymers. Unfortunately, only few papers related to the structure and properties of FEP/PTFE blends are found the in literature. The structure of such blends was investigated in [40,41,42,43,44]. Analysis of such blends using differential scanning calorimetry and X-ray diffraction shows that FEP and PTFE are immiscible and coexist as separate phases over a wide range of blend concentrations, and no co-crystallisation effect was observed [40,41,42,43]. Chemical bonds between these two fluoropolymers can form only as a result of crosslinking under irradiation [44]. Increasing the FEP content in PTFE-based blends results in a decrease in the viscosity of the blend melt and in an improvement of the dielectric properties and water absorption [42]. The addition of a small amount of PTFE to the FEP matrix allows one to improve the strain-hardening properties of FEP significantly [43]. Wear resistance studies were carried out for PTFE-based blends only. References [45,46] report that the addition of up to 40 wt.% FEP to PTFE allows one to increase the wear resistance by almost two orders of magnitude compared with pure PTFE. On the other hand, a wet wear study shows that addition of 20 wt.% of FEP to PTFE does not result in any positive effect because FEP cannot effective prevent the pulling out of PTFE crystalline bands duringwear [47]. It was observed [46] that a decrease in FEP filler size can significantly improve the tribological properties of PTFE-based blends. To check this effect in the case of FEP-based materials, in this study we used two types of PTFE fillers with different particle size.

To provide self-lubrication properties to composites the addition of solid lubricants is required. The most commonly used solid lubricants are graphite [48,49], boron nitride [50,51], molybdenum disulfide [52,53] and tungsten disulfide [54]. As it was mentioned above, increased thermal conductivity is advised to improve the tribological properties of polymer composites; however, as it was found in [29], QC fillers do not allow one to increase the thermal conductivity of polymer composites significantly. Therefore, it is reasonable to use solid lubricants which can also increase the thermal conductivity of composites. Whereas the thermal conductivity of molybdenum and tungsten disulfides is not very high [55,56], both graphite and boron nitride have excellent thermal conductivity. As boron nitride is a relatively expensive material, in this study we used graphite fillers both to provide self-lubrication properties and increase the thermal conductivity of composites. Additionally, to evaluate the effect of graphite crystalline structure peculiarities on the composite behavior, we also used carbon black as filler for FEP-based composites.

## 2. Materials and Methods

### 2.1. Materials

**Figure 1:**
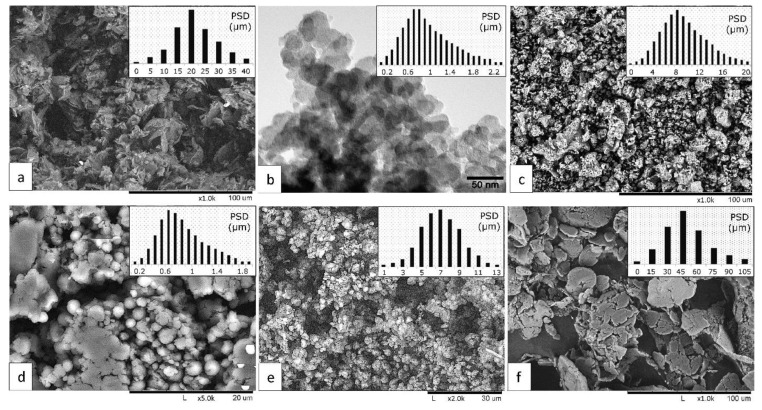
SEM images and particle size distribution of the initial powders: Graphite (**a**); Carbon black P-234 (**b**); quasicrystal Al_73_Cu_11_Cr_16_ (**c**); FEP (**f**); PTFE trade F-4PN (**d**); PTFE trade FORUM (**e**).

Scanning electron microscopy (SEM) images and particle size distribution histograms obtained by SEM images analysis of the initial powder matrix and fillers are shown in Figure 1. Fine grinding synthetic graphite (Gr) powder with an average particle size of 25 µm (GraphitEl—Moscow Electrode Plant, Moscow, Russia) was used as a thermally conductive and self-lubrication filler. Active furnace fine carbon black (CB) trade P-234 with an average particle size of 70 nm (Carbon Ltd, Ivanovo, Russia) was used as reinforcing filler.

Decagonal quasicrystalline (QC) Al_73_Cu_11_Cr_16_ powders were used as reinforcing and wear resistance filler. Quasicrystalline powder was obtained by mechanical alloying from the elemental powders for 120 min with subsequent annealing at 750 °C for 1 h; the content of decagonal quasicrystalline phase in powder was more than 96 vol. %. This powder also contains a small amount of Al_4_Cu_9_ crystalline phase. The average particle size of the quasicrystalline powders was 10 μm. The formation route and structure of this quasicrystalline powder were described in our previous paper [25].

**Figure 2:**
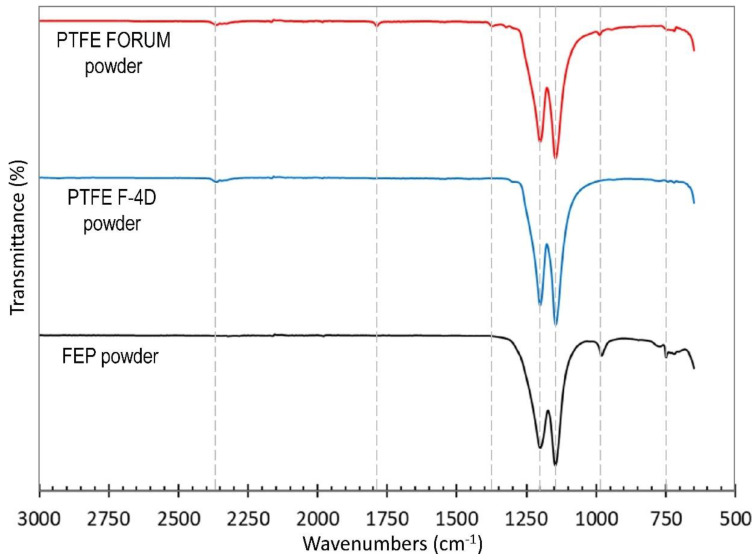
FTIR spectra of FEP, PTFE F-4D and nano PTFE FORUM powders.

Tetrafluoroethylene—hexafluoropropylene copolymer fluoroplastic FEP powder was used as a polymer matrix (HaloPolymer Kirovo-Chepetsk, LLC, Kirovo-Chepetsk, Russia). FEP is a finely dispersed spherical powder with an average particle size of 8 µm. Two types of PTFE fillers were used as a solid lubricant additive. The first one (designated as PTFE) was Fluoroplast-4 grade PN fully fluorinated PTFE powder with an average particle size of 50 µm (HaloPolymer Kirovo-Chepetsk LLC). The second one (designated as PTFE nano) was PTFE powder trademarked as Forum (Institute of Chemistry of the Far Eastern Branch of the Russian Academy of Sciences, Vladivostok, Russia). The powder has a spherical shape with the average particle size of 65 nm which can form aggregates. To study the initial polymer structures Fourier transform infrared spectrometry (FTIR) was carried out using a Nicolet 380 spectrometer (Thermo Scientific, Waltham, MA, USA; spectral range of 4000–450 cm^−1^, resolution of 1 cm^−1^). The FTIR spectra of FEP powder, PTFE F-4D powder, and PTFE FORUM powder are shown in Figure 2.

The FTIR spectra of FEP powder shows –CF_2_–CF_2_– bands at 1201.40 and 1146.45 cm^−1^ that are attributed to the stretching vibration of the –CF_2_ [56]. Also, FEP powder is characterized by a peak at 981.61 cm^−1^ that is assigned to the –CF_3_ stretching vibration of the hexafluoropropylene unit and a peak at 750.26 cm^−1^ characteristic of the CF–CF_3_ moiety. The FTIR spectra of PTFE F-4D powder shows the –CF_2_–CF_2_– bands at 1144.48 and 1200.20 cm^−1^. PTFE F-4D also shows the combination band for –CF_2_ stretching vibration at 2365 cm^−1^. The FTIR spectra of PTFE Forum powder was like PTFE F-4D FTIR spectrum (peaks at 1144.48, 1200.20, and 2365 cm^−1^). Besides those, PTFE Forum powder shows a weak peak at 1786 cm^−1^ (CF=CF_2_ groups) and peak at 750.26 cm^−1^ (side trifluoromethyl groups—CF_3_), which is related to the low-molecular weight fractions of FEP [57].

### 2.2. Composites Formation

The fillers were introduced into a FEP polymer matrix using a laboratory high energy planetary ball mill (HEBM) equipped with water cooling (APF-3, Institute of Solid-State Chemistry and Mechanochemistry SB RAS, Novosibirsk, Russia). The carrier rotation speed was 450 min^−1^, mixing duration was 70 min, steel balls of 6–9 mm in diameter were used as grinding bodies, steel vial volume was 900 mL (pair), the material load was 70 g, the ball to powder ratio was of 14:1. The list of the obtained FEP-based compositions is given in Table 1. All compositions were labeled as indicated for easy understanding of the reader.

Test samples were produced on a HAAKE MiniJet injection laboratory molding machine (Thermo Fisher Scientific, Karlsruhe, Germany) at a cylinder temperature of 380 °C, the injection pressure of 600 bar (time 25 s), mold temperature of 160 °C and post-pressure of 200 bar (time 60 s). Before being injection molded as-milled powder mixtures were dried at 140 °C for 1 h. We used two different injection molds. The first one is a cylindrical mold Ø12.7 mm × 2 mm and was used to obtain samples for thermal conductivity measurements. The second one is a plate 80 × 10 × 4 mm^3^, these plates were used to obtain samples for mechanical, thermo-mechanical, and tribological tests. Samples for tensile tests were cut from plates using hydraulic press (see Section 2.6 for details), samples for thermo-mechanical test were cut from plated by knife (see Section 2.8 for details). Samples for tribological tests were also cut from the plate and then isostatically hot pressed at 200 °C using special mold, see also Section 2.9. 

### 2.3. Characterization of the Samples Structures

We investigated the rapture surfaces of the FEP-based composite sample after tensile test, fracture surfaces (prepared by samples cracking in liquid nitrogen) by scanning electron microscopy (SEM) using a TM-1000 microscope (Hitachi Ltd., Tokyo, Japan).

### 2.4. Density Measurements

The densities of FEP-based composite samples were measured by hydrostatic weighing in distilled water and ethyl alcohol according to ISO 1183-1: 2019 (Plastics—Methods for determining the density of non-cellular plastics) using an AND GR 202 analytical balance (A&D Limited, Tokyo, Japan) equipped with a hydrostatic weighing AD-1653 accesory.

### 2.5. Hardness Measurements

The hardness of FEP-based composite samples on the Shore D scale was measured using TSh-D handheld Durometers (Novotest, Saint Petersburg, Russia) in accordance with ISO 868:2003 (Plastics and ebonite; determination of indentation hardness using a durometer (Shore hardness).

### 2.6. Mechanical Tests

The tensile test was carried out at room temperature according to ISO 527-2:2012 (Plastics—Determination of tensile properties) using a Zwick/Roell Z020 universal tensile testing machine (Zwick GmbH, Ulm, Germany) and a MultiXtens high-precision strain measurement system. Before testing all the samples were conditioned in accordance with ISO 291: 2008 (Plastics—Standard atmospheres for conditioning and testing) under a standard 23/50 atmosphere for 88 h. The test speed was 20 mm/min. The total number of specimens tested at one point in the tensile test was not less than 5. The samples for tensile tests are dumbbell of 75 mm length (type 1BA), they were cut from 80 × 10 × 4 mm^3^ molding samples on a hydraulic press.

### 2.7. Thermal Conductivity Tests

Thermal diffusivity was measured in the temperature range from 25 to 200 °C in accordance with ASTM E1461-07 (Standard Test Method for Thermal Diffusivity by the Flash Method) using the NETZSCH LFA447 NanoFlash device (Netzsch GmbH, Selb, Germany). The study was carried out using cylindrical specimens each having a 12.7 mm diameter and a thickness of 1–1.5 mm.

Specific heat capacities C_p_ of composite materials in the temperature range from 25 to 350 °C were measured using a NETZSCH DSC 204 Phoenix F1 differential scanning calorimeter (Netzsch GmbH) in accordance with ISO 11357-4: 2014 (Plastics—Differential scanning calorimetry (DSC)—Part 4: Determination of specific heat capacity). Sapphire was used as standard reference. The tests were carried out on pieces 5 mm in diameter and weighing 24–25 mg, in a protective argon atmosphere.

Thermal conductivity was calculated using the formula:

λ(t) = a(t)·d_k_·C_p_(t) (1)

where *λ*(t) is thermal conductivity coefficient at a certain temperature t, W/(m·K); a(t) is thermal diffusivity at certain temperature t, mm^2^/s; d_k_ is the material density, g/cm^3^; *C*_p_(t) is specific heat capacity, J/(g·K).

### 2.8. Thermo-Mechanical Tests

Vicat softening temperature (VST) was measured in accordance with ISO 306 with a CEAST HV3 tester (Instron, Norwood, MA, USA). Determination of the temperature at which a standard indenter (3 mm long, circular cross-section, and area 1 ± 0.015 mm^2^) penetrates 1 mm into the surface of the test specimen under the load when the temperature is raised at a uniform rate. The temperature at 1 ± 0.01 mm penetration is quoted as the VST. Method B 120, using a force of 50 N and heating rate of 120 °C/h. Test specimens were 4 mm thick and 10 mm square. The total number of specimens tested at one point in the tensile test was not less than 3.

### 2.9. Tribological Tests

**Figure 3:**
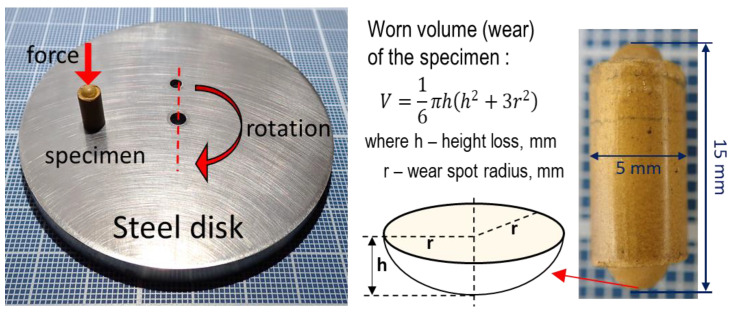
Pin-on-disk sliding wear test and determination of wear of the specimen.

Tribological tests of the produced molded materials were carried out according to the pin-on-disc regime on a Cetr UMT-3 friction machine (Bruker Corp., Billerica, MA, USA). Dry sliding wear tests were conducted at room temperature, with a load of 2 N, and a sliding speed of 0.5 m/s. The duration of each test was 30 min. The test pin specimen’s shape was cylinder Ø15 × 5 mm with two hemispheres to both ends (Figure 3). Thus, the contact pin with steel counterbody is a point contact and not a flat contact. The wear of the samples was calculated by the volume loss (V) of the material after 30 min of sliding using the following formula:

(2) $$V\;=\;\frac{1}{6}\pi h\left(h^{2}\;+\;3r^{2}\right)$$

where *h* is the height loss of the sample, mm

*r*—radius of the wear spot of the sample, mm

The height loss of the sample is determined by the tribological machine sensor during the test. The diameter of the wear spot of the samples was determined after 30 min by using an optical microscope equipped with a measuring scale with graduation of 0.05 mm. The counterbody was stainless steel 440 C disc Ø70 × 10 mm. Before each test, counterbody surface was polished with diamond paste to obtain a roughness of R_a_ = 0.08 μm. 

## 3. Results and Discussion

### 3.1. Thermal Analysis

The thermal diffusivity of the unfilled FEP and FEP-based composites was measured in the temperature range of 25–200 °C. The results of thermal diffusivity, density, and specific heat capacity of the unfilled FEP and FEP-based composites at 25 °C are presented in Table 2. QC filler has low thermal conductivity, therefore the presence of QC in a FEP matrix has a negligible effect on the thermal diffusivity of the material, insignificant increase in thermal diffusivity was found only for sample containing 10 wt.% QC (Table 2, no. 2–9), also Table 2 shows that addition of CB and PTFE has no effect on thermal conductivity value. On the contrary, introducing of graphite powder into FEP matrix leads to an increase of the thermal diffusivity in 2–3 times (Table 2, no. 10, 11). Table 2 also provides data on the density and heat capacity of composites required to calculate the thermal conductivity of a material in the temperature range of 25–200 °C. The density values were taken at 25 °C. DSC analysis measured the heat capacity of the material in a temperature range of 25–200 °C.

**Figure 4:**
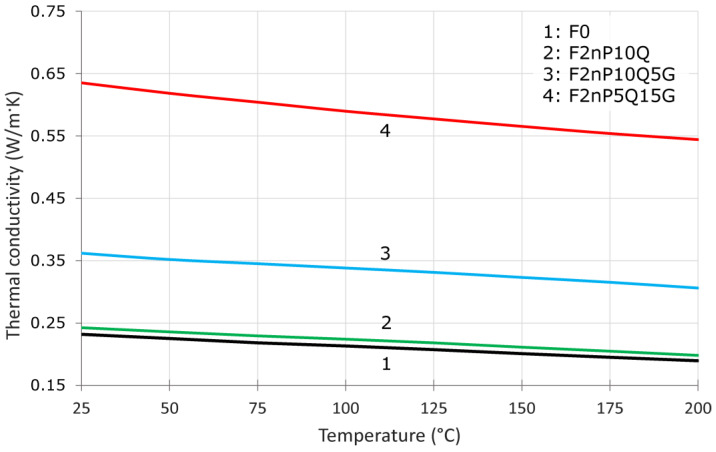
Temperature dependences of thermal conductivity for unfilled FEP and FEP-based composites.

Figure 4 shows the temperature dependences of the thermal conductivity of FEP-based composites between 25–200 °C. For all compositions, we can observe a linear decrease in the thermal conductivity of the material with increasing temperature. Introducing 10 wt.% QC Al_73_Cu_11_Cr_16_ (line 2) has almost no effect on the thermal conductivity of the material relative to FEP (line 1). The addition of 5 wt.% (curve 3) or 15 wt.% (curve 4) graphite powder has led to 1.5 or 2.5 times increase in the thermal conductivity of the FEP, respectively.

### 3.2. Mechanical and Thermo-Mecahnical Properties

The results of mechanical tests of the unfilled FEP and FEP-based composites are presented in Table 3. The presence of a small amount of QC and CB in FEP matrix (Table 3, no. 2) led to an increase in Shore D hardness from 57.5 to 59 units. Tensile properties of the composite are comparable to those of unfilled FEP. Introducing 5 wt.% of PTFE or nano-PTFE filler (Table 3, no. 3–6; 10) has a particularly negative effect on Shore D hardness and tensile elongation at break of the composite, which may indicate the formation of weakening agglomeration areas of PTFE particles in the FEP matrix. For composites containing 2 wt.% of nano PTFE, an increase in QC content from 2.5 to 10 wt.% (Table 3, no. 7–9) leads to an increase in the Shore D hardness from 57.5 to 61.5 units and in the tensile modulus of the material from 0.59 to 0.7 GPa. Introducing of QC has led to increase in the Vicat softening temperature (VST) of FEP-based composite from 83.7 to 97.2 °C. We can note that VST values is strongly associate with hardness and Young modulus of material, the higher is the hardness/modulus, the highest VST values were achieved.

**Figure 5:**
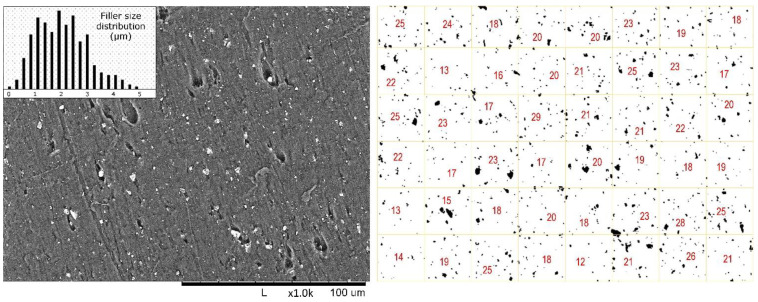
SEM image of fracture surface of F2nP10Q (obtained in liquid nitrogen) and its invert binary image with grid (a quantity of particles in each cell is depicted).

A SEM image of the fracture surface of the F2nP10Q specimen, obtained by sample breaking in liquid nitrogen, and its inverted binary image presented in Figure 5. Filler particle size distribution is 0.5–4.5 µm (measured from SEM image). Filler distribution in FEP matrix was studied using an inverted binary SEM image with grid of F2nP10Q. The particle quantity was calculated in each cell, the average particle number in sell was of 20 ± 4. Analyzing presented inverted binary image, we may sign that using HEBM technique to prepare composite provided a uniform distribution of filler in polymer matrix.

**Figure 6:**
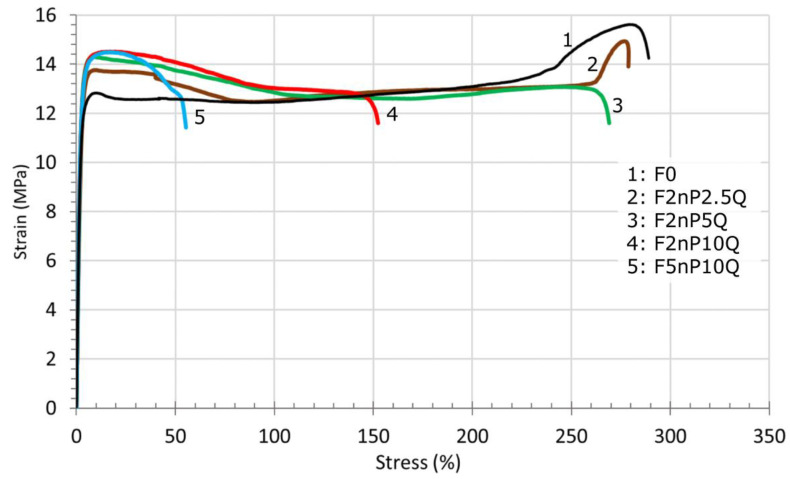
Tensile stress-strain curves of unfilled FEP and FEP-based composites.

Tensile stress-strain curves for unfilled FEP and FEP/PTFE-nano/QC Al_73_Cu_11_Cr_16_ composites are presented in Figure 6. The tensile behavior of unfilled FEP (curve 1) is common for ductile, semi-crystalline thermoplastic with large deformations under tension before local failure. The curve 1 includes the following regions: Elastic (0–2.5%)—Yield (2.5–12.5%)—Necking (12.5–25%)—Cold drawing (25–200%)—Strain hardening (200–280%)—Fracture. Necking region of unfilled FEP poorly revealed because of the specimen goes thinner in a very uniform manner, rather than forming a ‘neck’. The tensile curve of F2nP2.5Q (curve 2) includes the same regions as for unfilled FEP with following transformations. Appearing of pronounced yield drop (the difference between the upper yield point and neck tension), broadening of Necking region (12.5–75%), and a remarkable narrowing in strain hardening region (260–280%). 

The rise of the yield drop is mainly because of the increase in the material’s stiffness with the addition of rigid QC Al_73_Cu_11_Cr_16_ particles. Necking is a local reduction in cross-sectional area at a point along the length of the sample. Broadening of necking region reveals an increase inhomogeneity in the composite structure upon the addition of particulate fillers, which induces the growth of internal local stresses. The tensile curve of F2nP5Q (curve 3) varies from F2nP2.5Q composite and pure FEP: the yield drop increases, widening of the necking region (12.5–120%), and the strain hardening is disappearing. The ultimate tensile strength is reached after passing the yielding region. Strain hardening is principally a consequence of chain orientation. Molecules are aligned parallel to the stretching direction in the cold drawn regions of both amorphous and crystalline areas of FEP. The disappearance of strain hardening may show a significant change in the molecular structure of the FEP. F2nP10Q composite exhibited an approximately 2-fold reduction in the material’s plasticity (curve 4). Compared to F2nP5Q (curve 3), the cold drawing region narrowed strongly (115–140%). For the F5nP10Q composite, fracture of the material occurs immediately after yielding (curve 5). The plasticity of the F5nP10Q decreases in 6 times, which reveals a high level of structure defects. 

**Figure 7:**
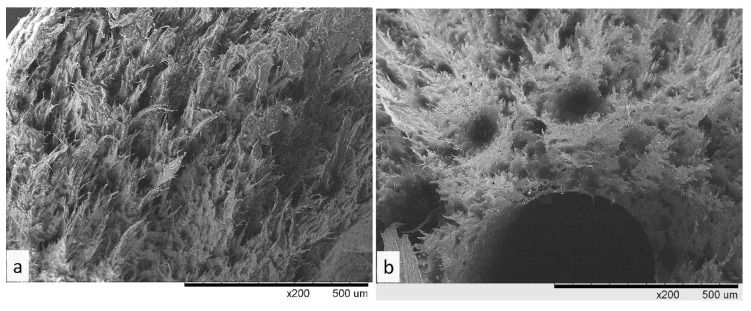
SEM images of the rupture surface after tensile test of F2nP10Q (**a**) and F5nP10Q (**b**).

We explore the elevated porosity of the F5nP10Q upon visual inspection of injection molding samples. This is confirmed by the SEM images of the rupture surface of samples F2nP10Q and F5nP10Q after tensile tests (Figure 7). The rupture surface of F2nP10Q sample includes a fibrillar structure generated by uniaxial deformation of the sample during tensile test. Rupture surface of F5nP10Q sample possesses a considerably less pronounced fibrillar structure (brittle fracture behavior) and contains large voids (300–500 µm). 

### 3.3. Tribological Test Results

The results of tribological tests on the wear and friction coefficient of the FEP-based composites after 30 min of dry sliding are given in Table 4. Unfilled FEP showed extremely high wear (1 mm^3^) and a high coefficient of friction (0.36). Introducing a small amount (1.25–2.5 wt.%) of QC Al_73_Cu_11_Cr_16_ filler leads to a wear reduction by 16–20 times (no. 2–4; 7) and also reduces the friction coefficient (0.27) of FEP. With an increase in filler loading of QC Al_73_Cu_11_Cr_16_ to 5–10 wt.% the wear of composite materials decreased by 50–65 times (Table 4, no. 5; 6; 8; 9) relative to unfilled FEP, but the coefficient of friction rises. Solid-lubricating PTFE additives of different types were added into FEP matrix to enhance the sliding properties of the FEP-based composite. We can confirm that nano-PTFE filler is more effective at reducing friction coefficients (Table 4, no. 8; 9) than regular PTFE filler (no. 5; 6). With an increase of QC Al_73_Cu_11_Cr_16_ filler in the concentration interval of 2.5–10 wt.%, the average coefficient of friction of FEP/PTFE nano/QC Al_73_Cu_11_Cr_16_ remaines at the same level (0.24–0.25). While using regular PTFE powder, the coefficient of friction of FEP/PTFE/QC Al_73_Cu_11_Cr_16_ increases from 0.25 to 0.28. It should be mentioned that the positive effect of nano-PTFE is achieved at a low degree of filling (2 wt.%), which does not negatively alter the mechanical properties of the composite material, whereas introducing 5 wt.% PTFE leads to a considerable drop in the elongation at break of the composite, as showed by tensile tests. 

**Figure 8:**
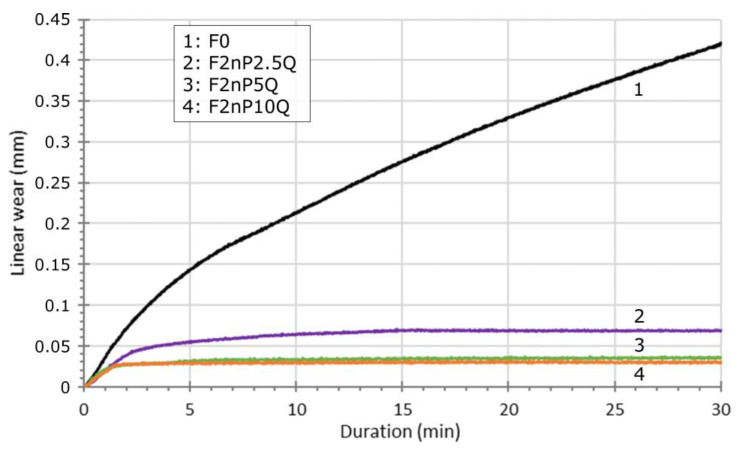
Time dependence of linear wear for unfilled FEP and FEP-nano PTFE-QC Al_73_Cu_11_Cr_16._

Figure 8 shows the time dependence of linear wear for FEP and FEP-nano PTFE-QC Al_73_Cu_11_Cr_16_ composites. The linear wear rate is the slope of the tangent at any point on a curve. Wear curves included a two segments: I—running-in stage, II - steady-state. For an unfilled FEP at the running-in stage, an extremely high wear rate is observed (6.5 × 10^−4^ mm/s), then reduced to 3.5 × 10^−4^ mm/s within 5 min of sliding. In the time interval of 5–30 min, the reduction is significantly slowed (from 3.5 × 10^−4^ to 1.2 × 10^−4^ mm/s), but the value of wear rate after 30 min of sliding remains at a high level. For composite containing 2.5 wt.% of QC (curve 2) the initial wear rate is reduced by 2 times and more (3.3 × 10^−4^ mm/s) related to unfilled FEP, which then rapidly decreases to 1.5 × 10^−4^ mm/s for the next 2 min of sliding. After 10 min of sliding, the wear rate of F2nP2.5Q composite approaches a zero value. As a result, the volume wear of the F2nP2.5Q after 30 min of sliding is considerably smaller (0.057 mm^3^) than that of pure FEP (1 mm^3^). With an increase in the concentration of QC up to 5 wt.% (curve 3) a further decrease in the wear rate at the very early stage of sliding (from 3.3 × 10^−4^ to 2.1 × 10^−4^ mm/s) was observed, and the volume wear of the F2nP5Q sample was found to be of 0.02 mm^3^. With a further increase in the QC content to 10 wt.% the wear rate curve does not undergo notable changes (curve 4). A gradual reduction in wear rate with increasing time occurrs due to the increase in contact area as the wear scar diameter increases. Thus, the addition of the QC filler into a FEP matrix results in the quick formation (due to reduction of the running-in period) of a stable wear scar (reduce running-in stage) with a smaller diameter (1 mm) than the unfilled FEP (2.6 mm). 

**Figure 9:**
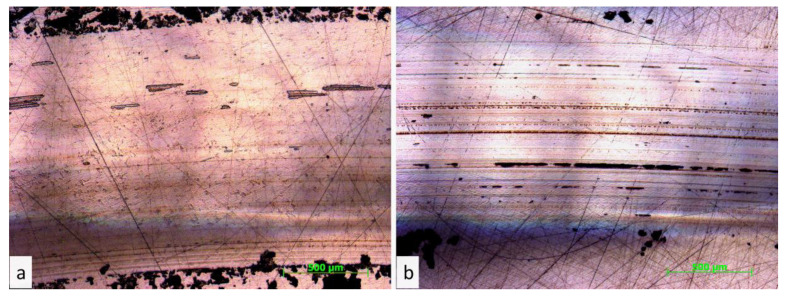
Optical images of steel counterbody surfaces after 30 min of dry sliding contact with. unfilled FEP (**a**) and FEP/1.25 wt.% CB/1.25 wt.% QC Al_73_Cu_11_Cr_16_ (**b**).

The proposed mechanisms for enhancing the wear resistance of FEP with the addition of QC Al_73_Cu_11_Cr_16_ can be as follows. The first basic explanation is that upon friction contact FEP with the steel counterbody a rigid QC Al_73_Cu_11_Cr_16_ filler has excellent load-carrying capacity, which restricts the wear and damage of the soft FEP matrix. The second mechanism may consist of the formation of FEP/QC Al_73_Cu_11_Cr_16_ stable frictional transfer film on the counterbody surface [32], which protects the composite from the rigid asperities of the steel counterbody. This explanation is confirmed by the analysis of optical images of the steel counterbodies after contact with an F0 and F1.25C1.25Q composite (Figure 9). The wear life of the pure FEP is very limited because FEP cannot form a durable transfer film on the steel counter body. FEP has been developed to form a big flake wear debris during the friction process. For F1.25C1.25Q composite the friction transfer layer on the surface of the counterpart can be observed. 

**Figure 10:**
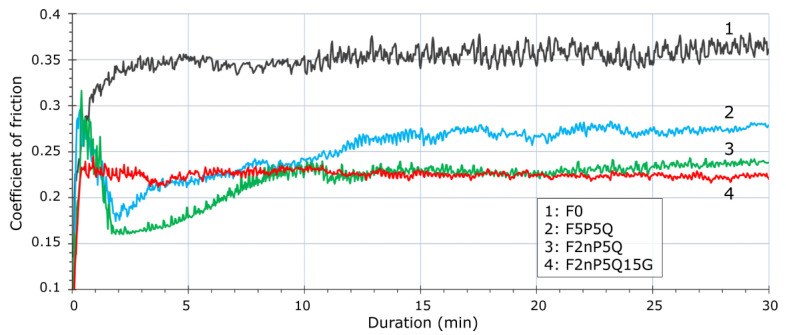
Time dependence of friction coefficient for unfilled FEP and FEP-based composites.

Figure 10 shows the variation of friction coefficient with time for unfilled FEP and FEP-based composites, and the influence of solid lubricants (PTFE, graphite) on the friction coefficient magnitude. With pure FEP, the friction coefficient increases rapidly in the initial stage of sliding, then it reaches a plateau (0.36) after 5 min of sliding, which corresponds to the transformation in wear from a high rate to a low rate. Also, it can be recognized an elevated level of friction noise on the graph. Latter can be attributed to a poor sliding performance of unfilled FEP. For FEP/PTFE/QC Al_73_Cu_11_Cr_16_ or FEP/PTFE-nano/QC Al_73_Cu_11_Cr_16_ composites, a similar sliding behavior is observed: a rapid increase in the coefficient of friction to 0.3 in the initial stage of sliding (0.5 min), followed by a rapid decrease to 0.18 (1.5 min). Then, there is a steady growth in the friction coefficient followed by a plateau. 

Differences in the effect of solid lubricating PTFE additives on the composite wear behavior were observed. For composites containing nano-PTFE the plateau occurs 5 min earlier, and the value of the friction coefficient is lower (0.24) than for composites contained coarse PTFE filler (0.27). This can be attributed to a better distribution of nano- PTFE particles than regular PTFE in the FEP matrix during the manufacturing process, and a more effective distribution on the counterbody surface during the running-in stage of sliding. One of the key features of nano-PTFE powder is its ability to quick cover the contact surface. Apparently, this explains the sharp decrease in the friction coefficient to 0.16 seen for the F2nP5Q composite during the running-in stage. The subsequent increase in the friction coefficient can be explained by a gradual rise in the concentration of QC Al_73_Cu_11_Cr_16_ particles in the contact region as the wear scar develops. The addition of 15 wt.% of finely dispersed graphite powder leads to a quick stabilization of the friction coefficient at the running-in stage, and the plateau at a friction coefficient of 0.23 occurs after 0.5 min of sliding in this case.

## 4. Conclusions

- Self-lubricating wear-resistant FEP/2 wt.% PTFE-nano/5 wt.% QC Al_73_Cu_11_Cr_16_ composite with excellent mechanical properties was produced by ball milling and injection molding;
- Addition of 5 wt.% fine dispersed QC Al_73_Cu_11_Cr_16_ powder enhances the wear resistance of FEP by 50 times in dry sliding wear;
- Addition of 2 wt.% nano-PTFE filler allows for a more efficient reduction of the friction as compared to regular PTFE without degradation of the plasticity of the composite;
- Addition of 15 wt.% fine graphite powder enhances the thermal conductivity up to 0.65 W/mK and also enhances the antifriction properties.

## Figures and Tables

**Table 1:** The list of the obtained FEP-based compositions.

Composite	Components Content (wt.%)	Label
FEP unfilled	100	F0
FEP-CB-QC Al_73_Cu_11_Cr_16_	97–1.25–1.25	F1.25C1.25Q
FEP-nano PTFE-QC Al_73_Cu_11_Cr_16_	95.5–2–2.5	F2nP2.5Q
	93–2–5	F2nP5Q
	88–2–10	F2nP10Q
	85–5–10	F5nP10Q
FEP-nano PTFE-QC Al_73_Cu_11_Cr_16_-Gr	83–2–10–5	F2nP10Q5G
	78–2–5–15	F2nP5Q15G
FEP-PTFE-QC Al_73_Cu_11_Cr_16_	93.75–5–1.25	F5P1.25Q
	92.5–5–2.5	F5P2.5Q
	90–5–5	F5P5Q
	85–5–10	F5P10Q

**Table 2:** Thermal properties of the unfilled FEP and FEP-based composites at 25 °C.

No.	Material	Thermal Diffusivity (mm^2^/s)	Density (g/cm^3^)	Specific Heat (J/g·K)
1	F0	0.107 ± 0.007	2.13 ± 0.004	1.06
2	F1.25C1.25Q	0.105 ± 0.003	2.157 ± 0.001	1.05
3	F5P1.25Q	0.103 ± 0.007	2.161 ± 0.005	1.05
4	F5P2.5Q	0.108 ± 0.001	2.178 ± 0.001	1.05
5	F5P5Q	0.108 ± 0.002	2.207 ± 0.001	1.03
6	F5P10Q	0.114 ± 0.001	2.249 ± 0.002	1
7	F2nP2.5Q	0.102 ± 0.003	2.173 ± 0.005	1.05
8	F2nP5Q	0.105 ± 0.001	2.199 ± 0.002	1.03
9	F2nP10Q	0.106 ± 0.001	2.243 ± 0.006	1
10	F2nP10Q5G	0.19 ± 0.006	2.251 ± 0.002	0.89
11	F2nP5Q15G	0.299 ± 0.009	2.209 ± 0.003	0.96

**Table 3:** The results of mechanical and thermo-mechanical properties of the unfilled FEP and FEP-based composites.

No.	Material	Hardness (Shore D)	Young’s Modulus (GPa)	Tensile Strength (MPa)	Elongation (%)	VST (°C)
1	F0	57.5 ± 1	0.59 ± 0.01	15.4 ± 0.4	295 ± 11	83.7 ± 0.5
2	F1.25C1.25Q	59 ± 1	0.6 ± 0.01	15.4 ± 0.1	295 ± 9	−
3	F5P1.25Q	55 ± 1	0.55 ± 0.01	14.1 ± 0.1	136 ± 9	−
4	F5P2.5Q	56 ± 1	0.57 ± 0.01	14.7 ± 0.1	48 ± 4	82.9 ± 0.4
5	F5P5Q	57.5 ± 1	0.6 ± 0.01	14.9 ± 0.2	44 ± 2	84.2 ± 0.5
6	F5P10Q	60 ± 1	0.66 ± 0.01	15.4 ± 0.1	34 ± 2	86.8 ± 0.5
7	F2nP2.5Q	58.5 ± 1	0.61 ± 0.01	14.7 ± 0.4	294 ± 16	87.1 ± 0.6
8	F2nP5Q	59.5 ± 1	0.64 ± 0.01	14.3 ± 0.2	280 ± 10	91.2 ± 0.7
9	F2nP10Q	61.5 ± 1	0.7 ± 0.01	14.4 ± 0.2	147 ± 7	97.2 ± 0.7
10	F5nP10Q	52 ± 1	0.69 ± 0.01	13.6 ± 0.8	55 ± 5	−

**Table 4:** The results of wear and friction coefficient of the unfilled FEP and FEP-based composites after 30 min of dry sliding.

No.	Material	Wear (mm^3^)	Coefficient of Friction
1	F0	1.038 ± 0.189	0.36 ± 0.03
2	F1.25C1.25Q	0.064 ± 0.019	0.27 ± 0.02
3	F5P1,25Q	0.064 ± 0.009	0.25 ± 0.02
4	F5P2.5Q	0.062 ± 0.019	0.27 ± 0.01
5	F5P5Q	0.029 ± 0.015	0.28 ± 0.02
6	F5P10Q	0.013 ± 0.002	0.28 ± 0.02
7	F2nP2.5Q	0.057 ± 0.008	0.24 ± 0.01
8	F2nP5Q	0.02 ± 0.006	0.24 ± 0.01
9	F2nP10Q	0.015 ± 0.006	0.25 ± 0.01
10	F5nP10Q	0.024 ± 0.007	0.24 ± 0.01
11	F2nP5Q15G	0.013 ± 0.005	0.23 ± 0.01

## Data Availability

The data presented in this study are available on request from the corresponding author.
